# Hierarchical neighbor integration graph attention network for autism spectrum disorder diagnosis

**DOI:** 10.3389/fpsyt.2026.1846996

**Published:** 2026-07-20

**Authors:** Di Ma, Liling Peng, Li Zhang, Weikai Li, Xin Gao

**Affiliations:** 1 College of Information Science and Technology & Artificial Intelligence, Nanjing Forestry University, Nanjing, China; 2 Department of PET/MR, Shanghai Universal Medical Imaging Diagnostic Center, Shanghai, China; 3 School of Computer and Artificial Intelligence, Shandong Jianzhu University, Jinan, China

**Keywords:** autism spectrum disorder, functional brain network, functional magnetic resonance imaging, graph attention network, multi-order interactions

## Abstract

**Introduction:**

Early diagnosis of autism spectrum disorder (ASD) based on resting-state functional magnetic resonance imaging (rs-fMRI) is crucial for effective intervention and rehabilitation. Using rs-fMRI, functional brain networks (FBNs) are constructed to represent interactions among brain regions of interest (ROIs), and existing graph neural network–based methods, particularly Graph Attention Networks (GATs), have shown promise for FBN-based ASD diagnosis. However, most current approaches primarily aggregate ROIs through low-order pairwise interactions, while higher-order neighbors are incorporated only implicitly through increased network depth. This strategy often leads to over-smoothing of node representations and limits the capture of informative higher-order brain interactions.

**Methods:**

To address these challenges, we propose the Hierarchical Neighbor Integration Graph Attention Network (HiNIGAT), a general graph learning framework that explicitly models multi-order interactions in FBNs. Specifically, HiNIGAT introduces a multi-order attention mechanism that constrains each attention head to specialize in a distinct neighborhood order, enabling the model to capture brain interactions from local connectivity to global network structure. Furthermore, a bidirectional gated fusion strategy is proposed to adaptively integrate complementary information across multi-order features, facilitating effective local–global representation collaboration.

**Results:**

Experiments on the ABIDE-I dataset demonstrate the effectiveness of HiNIGAT.

**Discussion:**

The results highlight the importance of explicit multi-order integration for ASD diagnosis.

## 1 Introduction

Autism spectrum disorder (ASD) is a neurodevelopmental condition with rising prevalence among children. Resting-state functional magnetic resonance imaging (rs-fMRI), which uses blood-oxygenation-level-dependent (BOLD) signals to detect brain activity, has been widely used to investigate functional connectivity alterations and potential neuroimaging biomarkers associated with ASD (1–3). Using rs-fMRI, one can model the brain as a functional brain network (FBN), where nodes denote the brain regions of interest (ROIs) and edges denote the functional connectivity between different pairs of ROIs. Differences in FBNs between ASD patients and healthy controls (HCs) provide valuable insights into the neural mechanisms underlying ASD and make FBN-based investigations a powerful approach for diagnosing brain disorders. However, conventional studies often decompose FBN-based diagnosis into two stages, feature extraction and model prediction, leading to significant risks of error accumulation and issues of information fragmentation.

The development of deep learning, particularly graph neural networks (GNNs), has advanced end-to-end FBN-based diagnosis to address the above limitations (4–6). GNNs model complex graph structures, transmitting node information across edges to facilitate node feature aggregation via neural networks. Owing to their ability to leverage the topological properties of FBNs for automatic node feature learning and updating, combined with their superior performance and interpretability, GNNs are widely employed in brain network analysis. For example, Parisot et al. (7) first applied graph convolutional networks (GCNs) to ASD classification, achieving classification performance that significantly outperformed traditional machine learning methods. Li et al. (8) proposed brain graph neural network (BrainGNN) model that utilizes ROI-aware graph convolutional layers and ROI-selection pooling layers for neurological biomarker prediction, surpassing various fMRI-based analysis methods. Subsequent studies include multi-view GCN (9), multi-pattern GCN (10) and multi-modal GCN (11), broadening the scope of GCN-based FBN analysis. However, GCNs suffer from limited performance scalability due to their reliance on predefined edge weights. To solve this problem, Velickoviˇ c´ et al. (12) proposed a graph attention network (GAT) that introduces the attention mechanism to dynamically learn adaptive weights during feature aggregation. This adaptability greatly enhances node correlation capture and has been widely used in diagnosing ASD (13).

However, most existing studies simply utilize low-order direct functional connections within FBNs to mask the attention computation, overlooking the rich structural information potentially embedded in high-order interactions among ROIs (14, 15). For instance, brain regions belonging to the same functional module often participate in coordinated neural processing through indirect communication routes. Even when two brain regions lack direct connection, they can still influence each other via common intermediate neighbors, giving rise to higher-order interaction patterns that support cooperative information transmission. Therefore, incorporating such high-order interactions is critical not only for enriching the receptive field of nodes in graph learning but also for enhancing the interpretation of complex neural mechanisms underlying brain disorders. Most existing approaches attempt to incorporate higher-order information indirectly by increasing model depth, relying on repeated message passing or implicit aggregation mechanisms. As a result, neighborhood information from different topological orders is entangled into a single representation space without explicitly distinguishing their structural roles. Such order-agnostic designs hinder the model’s ability to preserve hierarchical relational patterns inherent in FBNs (16, 17). Consequently, rather than merely expanding neighborhood coverage through deeper message passing, addressing this challenge requires an order-aware framework that explicitly distinguishes neighborhood information across multiple topological orders and integrates complementary representations in a structured and adaptive manner.

In this work, we propose the Hierarchical Neighbor Integration Graph Attention Network (HiNIGAT), which explicitly constructs order-specific sparsity-controlled and reliability-aware neighborhood structures as structural priors to stably model multi-order interactions in FBNs. Based on these hierarchical neighbors, HiNIGAT introduces a dedicated multi-order attention mechanism that directly constrains each attention head to specialize in aggregating information from a distinct order neighborhood. Such a pseudo-multi-head design explicitly captures region interactions spanning from direct local connectivity to broader global network structures, enriching feature diversity and mitigating the over-smoothing problem associated with deep graph networks. Furthermore, recognizing the complementary nature of information captured at different orders, we introduce a bidirectional gated fusion strategy. This strategy adaptively integrates features from the multi-order attention heads along both bottom-up (local-to-global) and top-down (global-to-local) pathways, simulating neurobiological information processing principles. The gating mechanism dynamically modulates information flow, ensuring effective integration while preserving discriminative features from each order. Extensive experiments on the ABIDE-I dataset demonstrate the effectiveness of HiNIGAT and highlight the importance of explicitly modeling higher-order region interactions for robust ASD diagnosis using rs-fMRI-derived FBNs.

## 2 Materials and methods

### 2.1 Participants and data preprocessing

The ABIDE-I dataset used in this paper was drawn from the public Autism Brain Imaging Data Exchange I (ABIDE-I) database (18). It contains over 1000 subjects with rs-fMRI data and corresponding phenotypic information. For a fair comparison, we followed the preprocessing procedure of the Configurable Pipeline for the Analysis of Connectomes (C-PAC) (19), which mainly includes slice timing correction, motion realignment, intensity normalization, nuisance signal regression, band-pass filtering (0.01-0.1Hz) and registration of the functional MRI images to MNI152 standard anatomical space. After preprocessing and quality control, 871 subjects were selected for the experiment, including 403 subjects with ASD and 468 HCs from 17 sites. Specifically, we used the quality-checked preprocessed ABIDE-I subset provided by the Preprocessed Connectomes Project (PCP) and accessed via the Nilearn fetcha_bide_pcp interface. The screening was performed at the subject level based on public quality-control labels and phenotypic-data availability. No acquisition site was manually excluded. Subjects who failed the public MRI quality-control assessment or lacked required phenotypic information were excluded. Detailed demographic information for the five sites with the largest number of participants is provided in **Table 1**. In this study, we extracted time series data of brain regions from the preprocessed data using the Anatomical Automatic Labeling 116 (AAL-116) atlas (20). Moreover, we do not enforce a uniform length of the time series in order to maximize the preservation of the original information. Although sequence lengths varied across sites, it did not affect the subsequent calculation of the FBN in practice. Furthermore, we also conducted additional evaluation on the ADHD-200 dataset (21), and the corresponding preprocessing, subject-screening details, and demographic information are provided in the **Supplementary Material** and **Supplementary Table 3**.

**Table 1:** Demographic information of the ABIDE-I dataset.

Site	ASD		HC	
	Age (years)	Sex (M/F)	Age (years)	Sex (M/F)
NYU	14.8 ± 7.1	64/10	15.8 ± 6.2	72/26
UM	12.9 ± 2.5	38/9	15.4 ± 3.4	55/18
USM	23.6 ± 8.4	43/0	20.9 ± 8.3	24/0
UCLA	13.1 ± 2.4	42/6	12.7 ± 2.1	32/5
Leuven	17.0 ± 4.1	23/3	18.4 ± 5.0	26/4
All sites	17.1 ± 8.0	349/54	16.8 ± 7.2	378/90

The data used in this study were obtained from the publicly available ABIDE-I and ADHD-200 datasets. All data were anonymized and contained no protected health information. Ethical approval and informed consent were obtained at each participating site. As this study involved secondary analysis of de-identified data, no additional ethical approval was required, in accordance with relevant data usage agreements and the Declaration of Helsinki.

### 2.2 Overview of HiNIGAT

Given the rs-fMRI data of an individual participant, we construct a subject-specific functional brain network (FBN) for graph-based diagnosis. Specifically, the brain is parcellated into *N* regions of interest (ROIs), and each subject is represented as an undirected graph G=(V,E,H, S). Here V denotes the set of ROI nodes, E denotes the set of pairwise connections between ROIs, $\text{H}\in {\mathbb{R}}^{N\times d}$ denotes the node feature matrix, and $\text{S}\in {\mathbb{R}}^{N\times N}$ denotes the connectivity matrix used to define graph topology and connection-strength information. The concrete construction of H and S is described in the implementation details. Based on the subject-level graph G, the goal is to learn a discriminative graph representation for classifying the subject as either ASD or HC.

**Figure 1:**
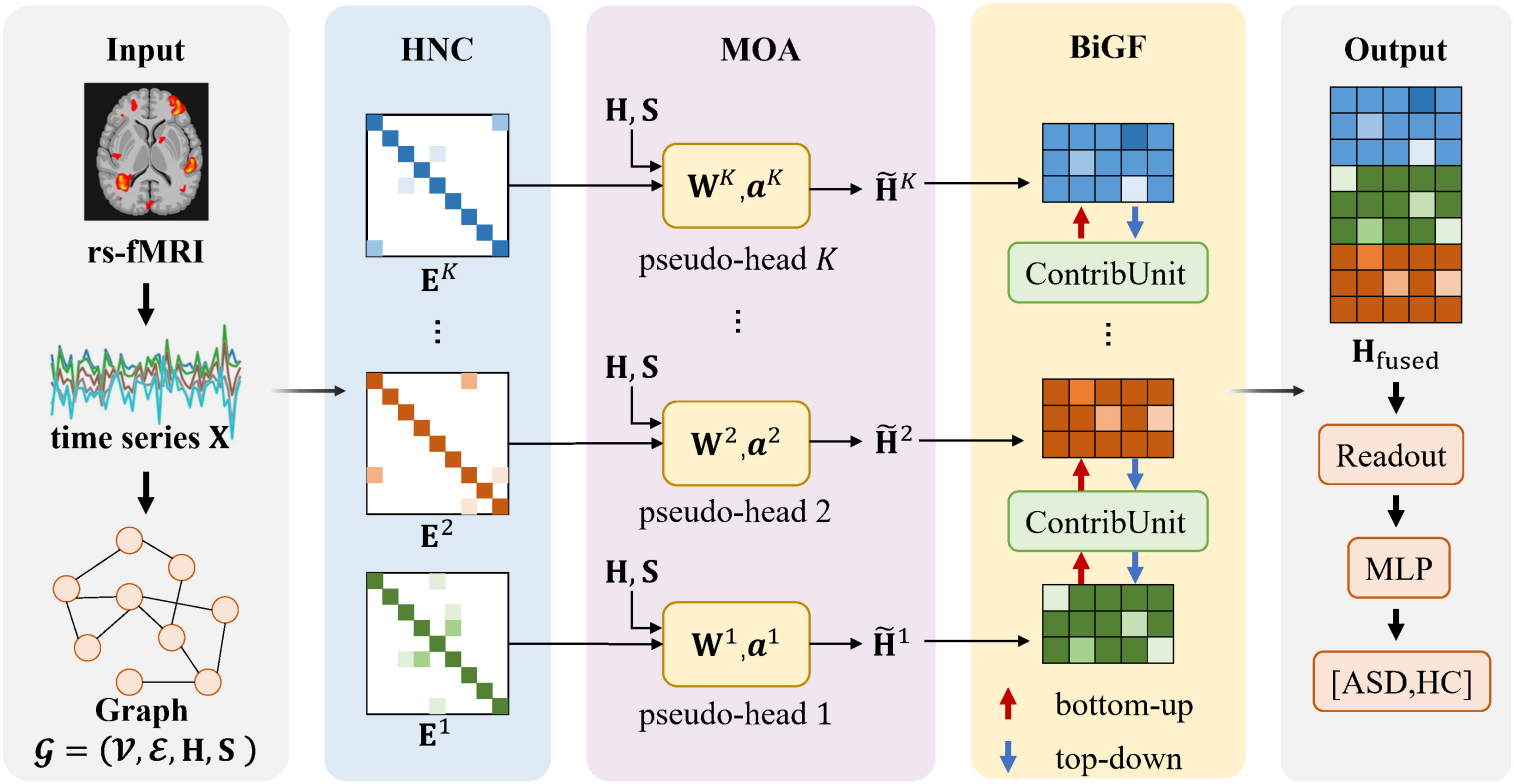
Overview of the proposed HiNIGAT framework. The rs-fMRI data are first preprocessed and converted into subject-specific functional brain networks. HNC constructs order-specific neighborhoods from the input FBN. MOA performs order-aware attention by assigning each pseudo-attention head to a specific neighborhood order. BiGF integrates the multi-order features through bottom-up and topdown gated fusion before graph-level ASD/HC classification. HNC, hierarchical neighbor construction; MOA, multi-order attention; BiGF, bidirectional gated fusion.

The overall architecture of HiNIGAT is illustrated in **Figure 1**. HiNIGAT consists of three main components, including hierarchical neighbor construction, multi-order attention, and bidirectional gated fusion. Starting from the subject-specific FBN, HiNIGAT first constructs a set of hierarchical neighborhoods to characterize functional interactions at different topological orders. Based on these order-specific structural priors, the multi-order attention (MOA) module assigns each pseudo-attention head to a predefined neighborhood order, so that node features can be aggregated in an order-aware manner. The bidirectional gated fusion (BiGF) module then progressively integrates the resulting multi-order representations along both bottom-up and top-down pathways to capture complementary contextual information across different orders. Finally, the fused graph representation is used for subject-level diagnosis.

In this way, HiNIGAT provides an end-to-end framework for hierarchical multi-order representation learning on functional brain networks. The detailed formulations of hierarchical neighbor construction, multi-order attention, and bidirectional gated fusion are presented in the following subsections.

### 2.3 Hierarchical neighbor construction

We construct hierarchical structural relations to explicitly model multi-order neighborhood information in FBNs, as formulated in Equations 1–5. Let P denote the binarized first-order adjacency matrix derived from the connectivity matrix S, and D the corresponding degree matrix. The *k*-order propagation matrix $P^{k}$ is defined as

(1) $${\text{P}}^{k}={\left({\text{D}}^{-1/2}\text{P}{\text{D}}^{-1/2}\right)}^{k}$$

which characterizes the interaction strength propagated through *k*-hop normalized walks.

Directly using high-order propagation matrices for graph construction is nontrivial, as deeper propagation tends to induce dense and excessively global relations while accumulating noise through repeated aggregation, which undermines sparsity and stability in FBN modeling. To this end, we construct orderspecific neighbors through a sparsity-controlled and stability-consistent mechanism. Specifically, we perform order-wise pre-selection by defining the *k*-th order candidate relations as

(2) $${\text{E}}_{\text{pre}}^{k}={\text{Top}}_{\rho_{\text{pre}}}({\text{P}}^{k}⊙(1-\overset{k-1}{\underset{t=1}{\text{V}}}{\text{E}}_{\text{pre}}^{t}))$$

where ∨ represents element-wise logical OR and $\rho_{\text{pre}}$ denotes a relative sparsity ratio that controls the number of retained relations at each neighborhood order. Specifically, for a graph with *N* ROIs, the operator Top*_ρ_*(·) retains the top d*ρN*(*N* − 1)*/*2e entries from the upper triangular part of the matrix, ensuring a sparsity constraint at each order. This formulation enforces strict order separation, such that each relation is uniquely associated with the first neighborhood order in which it appears, preventing redundant reuse of the same relation across multiple orders and reducing cross-order leakage. Rather than serving as a neurobiological assumption that functional interactions between ROIs are exclusive across orders, this separation is used as an operational graph construction strategy that allows MOA to receive more clearly organized order-specific signal sources.

To further assess the reliability of order-wise candidate relations, we refine the pre-selected structures through a stochastic measurement-based consistency criterion. Specifically, let ${\left\{{\text{Φ}}_{m}\right\}}_{m=1}^{M}$ denote a set of independent stochastic measurement operators, where each Φ*_m_* provides a compressed observation of the candidate structure. For each measurement operator, a measurement-domain response matrix is computed as

(3) $${\text{C}}_{m}^{k}={\text{Φ}}_{m}{\text{E}}_{\text{pre}}^{k}{\text{Φ}}_{m}^{T}$$

which characterizes how the *k*-th order candidate relations are perceived under the *m*-th stochastic observation. Such randomized measurements have been widely used to assess the robustness and reproducibility of structural patterns, as stable relations tend to remain observable across repeated stochastic measurements, while unstable ones are suppressed in expectation (22–24).

Since the response matrices are defined in the measurement domain, we further derive an edge-level consistency score by aggregating the induced responses of each candidate relation (*i,j*) across stochastic measurements:

(4) $${\text{score}}_{ij}^{k}=\frac{1}{M}\sum_{m=1}^{M}\langle \phi_{m,i},C_{m}^{k}\phi_{m,j}\rangle$$

where $\phi_{m,i}$ and $\phi_{m,j}$denote the *i*-th and *j*-th column vectors of Φ*_m_*. Larger values indicate relations that repeatedly induce non-negligible responses under stochastic projections, reflecting higher structural reliability.

Based on the resulting edge-level consistency scores, the refined structural relation matrix is then obtained by retaining the most reliable relations,

(5) $${\text{E}}^{k}={\text{Top}}_{\rho_{\text{tar}}}({\text{E}}_{\text{pre}}^{k}⊙{\text{score}}^{k})$$

yielding order-specific, sparsity-controlled and reliability-aware structural relations that serve as stable priors for subsequent multi-order attention and hierarchical fusion.

### 2.4 Multi-order attention module

Given the order-specific neighborhood structures E*^k^*, HiNIGAT introduces a Multi-Order Attention (MOA) module, defined in Equations 6–10, to explicitly model node interactions across different topological orders. Unlike conventional multi-head attention, MOA adopts a pseudo-head design, where each attention head is constrained to operate on a predefined order-specific neighborhood, thereby preventing premature crossorder mixing and enabling explicit disentanglement of local direct interactions and broader indirect interactions. This design does not imply that different orders are independent in the final representation, but rather preserves the distinguishability of signals from different topological scales before feature-level fusion.

Let h*_i_* denote the feature vector of node *i*, the unnormalized attention coefficient between node *i* and node *j* is computed as

(6) $$r_{ij}^{k}=\text{LeakyReLU}([{\text{h}}_{i}{\text{W}}^{k}\;\|\;{\text{h}}_{j}{\text{W}}^{k}]{\text{a}}^{k})$$

where ${\text{a}}^{k}\in {\mathbb{R}}^{2d^{\prime }}$ and ${\text{W}}^{k}\in {\mathbb{R}}^{d\times d^{\prime }}$ are the learnable parameters, [·‖·] denotes vector concatenation.

While the attention score $r_{ij}^{k}$ reflects data-driven semantic relevance, it does not account for connection strength information. Let $\left|s_{ij}\right|$ denote the connection strength between node i and node j derived from S. To incorporate this information in an order-aware manner, we combine it with the hierarchical neighbor structure by defining the order-specific connection strength prior $s_{ij}^{k}$ as

(7) $$s_{ij}^{k}=|s_{ij}|\cdot e_{ij}^{k}$$

where hierarchical neighbor construction determines which connections are activated at each order, while S provides the corresponding connection strength.

To integrate the connection strength prior into attention learning while controlling its influence on the attention logits, we inject it in a log-domain additive form with a learnable scaling coefficient *λ*:

(8) $${\tilde{r}}_{ij}^{k}=r_{ij}^{k}+\lambda \log\;(s_{ij}^{k}+\epsilon )$$

where ϵ is a small constant for numerical stability and *λ* is a sigmoid-parameterized learnable scalar constrained to (0,1) that adaptively controls the influence of the connection strength prior.

The final attention coefficient $a_{ij}^{k}$ is obtained by applying softmax over the valid *k*-th order neighborhood $j^{\prime }\in N_{i}^{k}$, which is determined by the order-specific structure *E^k^*:

(9) $$a_{ij}^{k}=\frac{\exp\;({\tilde{r}}_{ij}^{k})}{\sum_{j^{\prime }\in N_{i}^{k}}\exp\;({\tilde{r}}_{ij^{\prime }}^{k})}=\frac{\exp\;(r_{ij}^{k}){\left(s_{ij}^{k}+\epsilon \right)}^{\lambda }}{\sum_{j^{\prime }\in N_{i}^{k}}\exp\;(r_{ij^{\prime }}^{k}){\left(s_{ij^{\prime }}^{k}+\epsilon \right)}^{\lambda }}$$

This formulation incorporates the connection strength prior as a controllable multiplicative factor after softmax normalization. When *λ* approaches 0, the attention mechanism degenerates to semantic attention without this prior. When *λ* approaches 1, it corresponds to the unscaled multiplicative prior. By learning *λ*, the model can adaptively adjust its contribution and reduce over-reliance on the absolute magnitude of connectivity values.

Consequently, using the normalized attention coefficients, the node representation for the *k*-th order pseudo-head is updated as

(10) $${\tilde{\text{h}}}_{i}^{k}=\text{ELU}(\sum_{j\in N_{i}^{k}}a_{ij}^{k}{\text{h}}_{j}{\text{W}}^{k})$$

In contrast to GAT and GCN that are restricted to modeling the semantic similarity or functional topology respectively, MOA incorporates both sources of information, enabling joint evaluation of node importance from both connectivity strength and learned semantic relevance.

### 2.5 Bidirectional gated fusion module

To enhance feature collaboration within the layer, we perform step-wise bidirectional fusion along the multi-order attention heads. Inspired by the feedforward and feedback dual pathways in neuroscience for perception and cognition (25), we propose a Bidirectional gated fusion (BiGF) module, described in Equations 11–18, to explicitly model contributions of distinct information aggregation pathways and capture the synergistic effects among multi-order features.

Given the set of multi-order feature matrices ${\left\{{\tilde{\text{H}}}^{k}\right\}}_{k=1}^{K}$, we perform progressive fusion along two complementary directions, Bottom-Up (BU) Fusion and Top-Down (TD) Fusion. The BU pathway simulates the process from local details to global concepts. Fusion begins with the first order and progressively incorporates information from higher orders:

(11) $${\text{H}}_{bu}^{k}=\{\begin{array}{ll} {\tilde{\text{H}}}^{1}, & k=1,\\ {\text{H}}_{bu}^{k-1}+\text{Δ}{\text{H}}_{bu}^{k}, & k=2,3,\ldots ,K, \end{array}$$

(12) $$\text{Δ}{\text{H}}_{bu}^{k}=\text{ContribUnit}({\text{H}}_{bu}^{k-1},{\tilde{\text{H}}}^{k}),\;k=2,3,\cdots ,K$$

where ${\text{H}}_{bu}^{k}$denotes the fused feature corresponding to the *k*-th order of the BU pathway, and $\text{Δ}{\text{H}}_{bu}^{k}$ represents the incremental contribution injected by the *k*-th order feature. The BU pathway is initialized with the first-order representation and then recursively updated by incorporating higher-order features.

In contrast, the TD pathway operates in the reverse direction, modeling a top-down feedback process in which global contextual information is progressively propagated toward lower orders. Starting from the highest-order representation, the TD fusion is formulated as:

(13) $${\text{H}}_{td}^{k}=\{\begin{array}{ll} {\tilde{\text{H}}}^{K}, & k=K,\\ {\text{H}}_{td}^{k+1}+\text{Δ}{\text{H}}_{td}^{k}, & k=K-1,K-2,\ldots ,1, \end{array}$$

(14) $$\text{Δ}{\text{H}}_{td}^{k}=\text{ContribUnit}({\text{H}}_{td}^{k+1},{\tilde{\text{H}}}^{k}),\;k=K-1,K-2,\cdots ,1$$

where ${\text{H}}_{td}^{k}$ denotes the fused feature state corresponding to the *k*-th order of the TD pathway, and sup $\text{Δ}{\text{H}}_{td}^{k}$ represents the incremental contribution injected by the *k*-th order feature. The TD pathway is initialized with the highest-order representation and then recursively updated toward lower-order features.

The operator ContribUnit is a GRU-like gated residual unit that computes the incremental contribution of the current order:

(15) $$\begin{array}{ll} \text{ΔH} & =\text{ContribUnit}({\text{H}}_{prev},{\text{H}}_{curr})\\ =\text{G}⊙({\text{H}}_{curr}-{\text{H}}_{prev}){\text{W}}_{c} \end{array}$$

(16) $$\text{G}=\sigma \;({\text{W}}_{g}\cdot [{\text{H}}_{prev}\|{\text{H}}_{curr}])$$

where ${\text{H}}_{prev}$ denotes the fused feature from the previous step (i.e. ${\text{H}}_{bu}^{k-1}$ in the BU pathway or ${\text{H}}_{td}^{k+1}$ in the TD pathway), and ${\text{H}}_{curr}={\tilde{\text{H}}}^{k}$ denotes the current-order feature to be incorporated. The gating matrix G adaptively regulates the magnitude of the contribution. Based on the step-wise contributions obtained from both pathways, we obtain the representation of each order by integrating the bidirectional contributions with the original order-specific feature:

(17) $${\text{F}}_{k}={\tilde{\text{H}}}^{\text{k}}+\beta_{k}(\text{Δ}{\text{H}}_{bu}^{k}+\text{Δ}{\text{H}}_{td}^{k})$$

where *β_k_*is a learnable scaling coefficient that calibrates the overall contribution strength for the *k*-th order. This formulation preserves the intrinsic semantics of each order while embedding it into a globally consistent hierarchical context through bottom-up accumulation and top-down feedback.

Finally, the fused representation is obtained by concatenating the finalized order-specific features across all orders:

(18) $${\text{H}}_{fused}=\text{Concat}(F_{1},\cdots ,F_{K})$$

This bidirectional gated fusion strategy enables each order to contribute explicitly and adaptively, while leveraging complementary bottom-up and top-down flows to achieve effective cross-order feature collaboration.

### 2.6 Readout and classification

To accomplish the graph classification task, a readout operation in Equation 19 is applied along the node dimension to compress node-wise representations into a graph-level feature representation. We followed the readout layer in (8), which is defined as

(19) $$z=\text{mean}\;{\text{H}}_{fused}\;\|\;\text{max}\;{\text{H}}_{fused}$$

where mean and max denote global average pooling and global max pooling, respectively. Finally, the classification task is performed by a single-layer perceptron based on the output **z**.

## 3 Results

### 3.1 Implementation details

Our experiments were implemented in a Python environment using PyTorch. For each subject, an individual functional brain graph was constructed independently from that subject’s rs-fMRI data. Node feature matrix H was defined using Pearson correlation coefficients, and the connection matrix S was constructed using absolute partial correlation coefficients. The binarized first-order adjacency matrix P was obtained by retaining the top 10% strongest connections from S. Hierarchical neighbor construction was also performed independently for each individual graph. The pre-selection sparsity ratio *ρ*_pre_ and target sparsity ratio *ρ*_tar_ were predefined as 15% and 10%, respectively, in the main experiments. For each neighborhood order, candidate relations were ranked and retained within the corresponding graph according to within-subject propagation and consistency scores under the predefined sparsity settings. The stochastic measurement operators ${\left\{{\text{Φ}}_{m}\right\}}_{m=1}^{M}$ were predefined using Rademacher random projections and kept fixed across all repeated folds.

The proposed HiNIGAT model consists of a single HiNIGAT layer followed by the readout layer and a single-layer perceptron for classification. The HiNIGAT layer incorporates the multi-order attention module and the bidirectional gated fusion module to model hierarchical neighborhood interactions. The layer employs *K* = 4 pseudo-attention heads, each with a hidden dimension of *d* = 16.

Model optimization was performed using the AdamW optimizer (26) with an initial learning rate of 1×10^−3^ and a weight decay of 1×10^−3^. The model was trained for up to 150 epochs with a dropout rate of 0.5 applied to the attention coefficients and layer outputs. The mini-batch size was set to 16 subjects. These model and training hyperparameters were fixed before running the repeated cross-validation experiments and kept unchanged across all repeated folds.

Performance was evaluated using repeated site-grouped 10-fold cross-validation. Acquisition sites were used as grouping units, ensuring that subjects from the same site were not shared between the training and testing sets within each split. The site-grouped fold assignment was repeated five times with different random seeds. Classification performance was reported as the mean and standard deviation across folds in terms of accuracy, recall, precision, F1-score, and the area under the ROC curve (AUC). Wilcoxon signed-rank tests with Benjamini–Hochberg FDR correction were conducted over the repeated folds to compare HiNIGAT with competing methods. In addition, we conducted a leave-one-site-out evaluation on ABIDE-I as a supplementary site-wise generalization analysis, where each acquisition site was held out in turn as the test set and the remaining sites were used for training. The corresponding results are reported in the **Supplementary Material**.

### 3.2 Comparison with baseline methods

To evaluate the classification performance of the proposed HiNIGAT, we compared it with both traditional machine learning (ML) methods and graph neural network (GNN)-based methods on the ABIDE-I dataset. For the ML baselines, we included Support Vector Machine (SVM) with a radial basis function (RBF) kernel (27) and a two-layer Multi-Layer Perceptron (MLP) with 64 hidden units (28). Following the feature construction strategy in (18), the upper-triangular elements of the correlation matrix were vectorized as input features. Within each fold, a ridge classifier was then applied to the training partition to select the most discriminative connections, resulting in a final feature dimension of 2000.

For the GNN-based baselines, we compared HiNIGAT against GCN (7), GraphSAGE (29), BrainGNN (8), MixHop (30) and SIGN (31). To further examine the effect of message-passing depth, we also included variants of GAT (32) with different numbers of graph attention layers, denoted as GAT-2L, GAT-4L, and GAT-6L, corresponding to two, four, and six stacked GAT layers, respectively. Among these methods, MixHop and SIGN are representative high-order graph learning models designed to more fully exploit multi-hop structural information. For a fair comparison, the graph convolution, pooling, and MLP settings of all competing GNN models were configured to be consistent with those of HiNIGAT, so that the compared methods had similar trainable parameter scales and overall model capacity.

**Table 2** summarizes the classification performance of different methods on the ABIDE-I dataset. Overall, GNN-based approaches generally showed favorable performance compared with conventional ML baselines, indicating the advantage of graph representation learning for FBN-based ASD diagnosis. Among the compared methods, high-order graph models, including MixHop, SIGN, and HiNIGAT, generally showed stronger performance than low-order graph baselines. The depth-controlled GAT variants further show that simply increasing the number of message-passing layers is not sufficient to achieve comparable performance. As shown in **Table 2**, GAT-2L, GAT-4L, and GAT-6L achieve accuracies of 67.70%, 67.89%, and 66.86%, respectively, which do not close the performance gap with HiNIGAT. In particular, increasing the number of GAT layers does not consistently improve performance, as GAT-6L performs worse than GAT-4L in both accuracy and AUC.

**Table 2:** Performance comparison of various methods on the ABIDE-I dataset.

Method	Accuracy	Precision	Recall	F1-score	AUC
SVM	64.56 ± 5.74∗∗	63.37 ± 9.74∗∗	63.45 ± 12.73	62.25 ± 8.14∗∗	71.03 ± 7.86
MLP	61.65 ± 4.82∗∗	60.54 ± 8.69∗∗	57.08 ± 9.14∗∗	58.13 ± 6.27∗∗	66.69 ± 5.83∗∗
BrainGNN	65.06 ± 4.14∗∗	68.01 ± 11.01	53.50 ± 14.20∗∗	58.01 ± 9.48∗∗	65.10 ± 5.30∗∗
GCN	67.51 ± 4.63∗∗	67.56 ± 7.70	60.20 ± 17.98∗	62.05 ± 11.45∗∗	67.37 ± 6.76∗∗
GraphSAGE	68.67 ± 5.75	69.57 ± 10.65	59.84 ± 17.16∗∗	63.00 ± 11.26∗∗	67.59 ± 8.58∗∗
GAT-2L	67.70 ± 5.98∗∗	67.08 ± 11.46	63.78 ± 15.01∗	64.20 ± 9.55∗∗	67.97 ± 7.08∗∗
GAT-4L	67.89 ± 6.01∗∗	64.96 ± 7.80∗∗	66.72 ± 21.69	63.97 ± 14.96	67.16 ± 7.93∗∗
GAT-6L	66.86 ± 4.31∗∗	68.24 ± 11.59	62.91 ± 22.74	61.35 ± 15.53∗	66.87 ± 6.76∗∗
MixHop	69.11 ± 4.58	68.20 ± 7.60	65.73 ± 13.54	66.03 ± 7.89	69.22 ± 5.29
SIGN	69.05 ± 5.33	71.57 ± 11.71	60.93 ± 18.88∗	63.11 ± 13.25∗	69.03 ± 7.27∗
HiNIGAT	70.30 ± 5.60	69.34 ± 9.41	68.65 ± 15.60	67.61 ± 9.32	71.20 ± 5.53

The best result in each column is highlighted in bold. ^∗^ and ^∗∗^ indicate that HiNIGAT significantly outperforms the corresponding method on the same metric according to Wilcoxon signed-rank tests with Benjamini–Hochberg FDR correction, with *q <* 0.05 and *q <* 0.01, respectively.

Notably, HiNIGAT achieved the best accuracy of 70.30%, recall of 68.65%, F1-score of 67.61%, and AUC of 71.20%. Compared with the strongest competing high-order graph baselines, HiNIGAT improved accuracy by 1.19% over MixHop and by 1.25% over SIGN. In addition, HiNIGAT achieved the best recall, F1-score, and AUC among all compared methods, demonstrating a more balanced classification capability. The significance markers in **Table 2** further indicated that HiNIGAT achieved statistically significant improvements over most conventional and GNN-based baselines on accuracy, F1-score, and AUC under the FDR-adjusted Wilcoxon signed-rank test, while the differences against several strong high-order baselines were more moderate.

Taken together, these results suggest that explicitly modeling order-specific neighborhood structures, together with multi-order attention and bidirectional hierarchical fusion, provides more discriminative graph representations for ASD diagnosis.

### 3.3 Ablation study

To thoroughly evaluate the contribution of each component in HiNIGAT, we conducted ablation studies on the ABIDE-I dataset under the same experimental settings as the full model. The analysis focused on two primary design strategies: replacing the multi-order attention (MOA) module with standard multihead attention (MHA), and replacing the bidirectional gated fusion (BiGF) module with simple feature concatenation. For a more fine-grained analysis, we further performed detailed component ablations under each strategy. The compared variants are summarized in **Table 3**.

**Table 3:** Ablation study of HiNIGAT on the ABIDE-I dataset.

Model	Accuracy	Precision	Recall	F1-score	AUC
w/o MOA	66.85 ± 5.77	64.48 ± 8.57	64.40 ± 15.94	63.15 ± 9.18	66.09 ± 6.88
w/o HO	68.64 ± 6.35	66.07 ± 9.27	66.18 ± 17.13	64.82 ± 10.86	68.67 ± 6.58
w/o OS	67.82 ± 5.13	67.02 ± 11.01	63.29 ± 17.47	63.05 ± 10.58	67.16 ± 7.48
w/o SOS	68.72 ± 5.68	65.74 ± 8.39	66.50 ± 15.87	65.05 ± 10.52	69.59 ± 6.42
w/o TW	68.28 ± 5.02	67.57 ± 10.07	60.22 ± 18.50	61.80 ± 13.19	68.26 ± 6.63
w/o BiGF	68.09 ± 6.38	66.26 ± 10.90	63.47 ± 19.52	62.98 ± 12.71	68.51 ± 6.58
w/o BU	67.97 ± 5.06	66.26 ± 12.35	59.05 ± 18.77	60.97 ± 14.32	67.91 ± 7.38
w/o TD	68.69 ± 6.11	67.89 ± 11.61	61.88 ± 19.98	62.51 ± 13.95	68.12 ± 7.20
HiNIGAT	70.30 ± 5.60	69.34 ± 9.41	68.65 ± 15.60	67.61 ± 9.32	71.20 ± 5.53

Specifically, the following variants were evaluated against the full HiNIGAT model:

- w/o MOA: replaces the MOA mechanism with standard MHA. Each attention head operates only on the first-order neighbors N*_i_*^1^ from a purely semantic perspective.
- w/o HO: removes high-order neighborhood information and uses only first-order neighborhoods while retaining the topological weighting scheme. This variant evaluates whether high-order neighborhood information provides additional benefit beyond direct functional connectivity.
- w/o OS: removes order separation in MOA, such that all attention heads operate on the union of multi-order neighbors.
- w/o SOS: replaces strict order separation with a soft order-wise weighting strategy, allowing a relation to be softly shared across multiple neighborhood orders.
- w/o TW: removes the topological weighting scheme from the aggregation function in MOA.
- w/o BiGF: replaces the bidirectional gated fusion mechanism with direct feature concatenation.
- w/o TD: removes the top-down fusion pathway from BiGF.
- w/o BU: removes the bottom-up fusion pathway from BiGF.

As shown in **Table 3**, the variant w/o MOA exhibits a clear performance decline, with accuracy decreasing from 70.30% to 66.85%, corresponding to a drop of 3.45% relative to the full model. This result indicates that the hierarchical neighbor matrices provide essential interaction priors that are effectively exploited by MOA. It also highlights the importance of high-order interactions as complementary information to low-order neighbors for capturing complex relationships among brain regions, which cannot be achieved by standard MHA alone.

For a more fine-grained analysis of MOA, the w/o HO variant shows that when only first-order direct functional connectivity is used, the accuracy decreases to 68.64%, with a drop of 1.66% compared with the full model. The AUC also decreases from 71.20% to 68.67%. This indicates that first-order information alone is insufficient to capture complementary high-order interaction patterns.

The w/o OS variant leads to a performance degradation of 2.48% in accuracy, indicating that simply merging multi-order neighbors is not sufficient. Premature cross-order mixing may weaken the distinguishability between direct low-order connectivity and indirect high-order contextual relations, thereby increasing subspace redundancy and making high-order contextual information less distinguishable from dominant low-order relations. Explicitly isolating information from different neighborhood orders enables more discriminative and diverse feature learning, thereby improving the effectiveness of multi-order representation learning.

The w/o SOS variant achieves an accuracy of 68.72% and an AUC of 69.59%, outperforming w/o OS on most metrics. This indicates that soft order-wise weighting can partially alleviate fully order-agnostic neighbor mixing. However, it remains lower than the full HiNIGAT model, with decreases of 1.58% in accuracy and 1.61% in AUC. This suggests that soft assignment may still permit strong low-order relations to dominate across multiple orders, whereas strict order-specific construction encourages different pseudo-attention heads to focus on their corresponding order-specific signal sources. Therefore, these results suggest that strict separation may provide a more effective computational organization for reducing redundant cross-order propagation and preserving high-order contextual information.

The variant w/o TW also reduces performance, with accuracy decreasing to 68.28%, corresponding to a drop of 2.02%, indicating that integrating topological information with semantic relevance benefits classification by emphasizing more informative connections. Taken together, these results demonstrate that MOA benefits not only from the inclusion of high-order neighborhoods, but also from explicitly organizing multi-order information through order-specific attention and connectivity-aware weighting.

The variant w/o BiGF causes a moderate performance degradation, with accuracy decreasing from 70.30% to 68.09%, underscoring the necessity of hierarchical feature fusion. Further analysis shows that the bottom-up (BU) pathway plays a relatively more critical role than the top-down (TD) pathway. Specifically, removing BU decreases accuracy to 67.97%, corresponding to a drop of 2.33%, while removing TD decreases accuracy to 68.69%, corresponding to a drop of 1.61%. This suggests that the BU pathway, which progressively propagates refined local representations toward higher-order contextual modeling, is particularly important for maintaining effective hierarchical representations.

Overall, HiNIGAT consistently outperforms all ablated variants across the major evaluation metrics. These results support the effectiveness of each proposed component and suggest that the combination of multi-order attention and bidirectional hierarchical fusion enables more comprehensive modeling of rich and complementary information in functional brain networks.

### 3.4 Parameter sensitivity analysis

To investigate the impact of sparsity in hierarchical neighbor construction (HNC), we conducted a sensitivity analysis while keeping all other settings fixed. Since higher-order neighbors are recursively derived from the first-order adjacency, altering first-order sparsity would fundamentally change the underlying graph topology and confound sparsity sensitivity with variations in graph construction. We therefore fixed the first-order connectivity to preserve a stable low-order backbone across all settings.

HNC involves two sparsity-related parameters, *ρ_pre_*for order-wise candidate pre-selection and *ρ_tar_*for determining the final sparsity of refined higher-order neighbors. In the following sensitivity analysis, we focus on *ρ_tar_*, as it directly controls the sparsity of the final hierarchical structures used for model learning, while *ρ_pre_*is fixed to provide a stable candidate pool. Higher-order neighbors are then progressively introduced under sparsity constraints ranging from 3% to 15% in our experiments.

**Figure 2:**
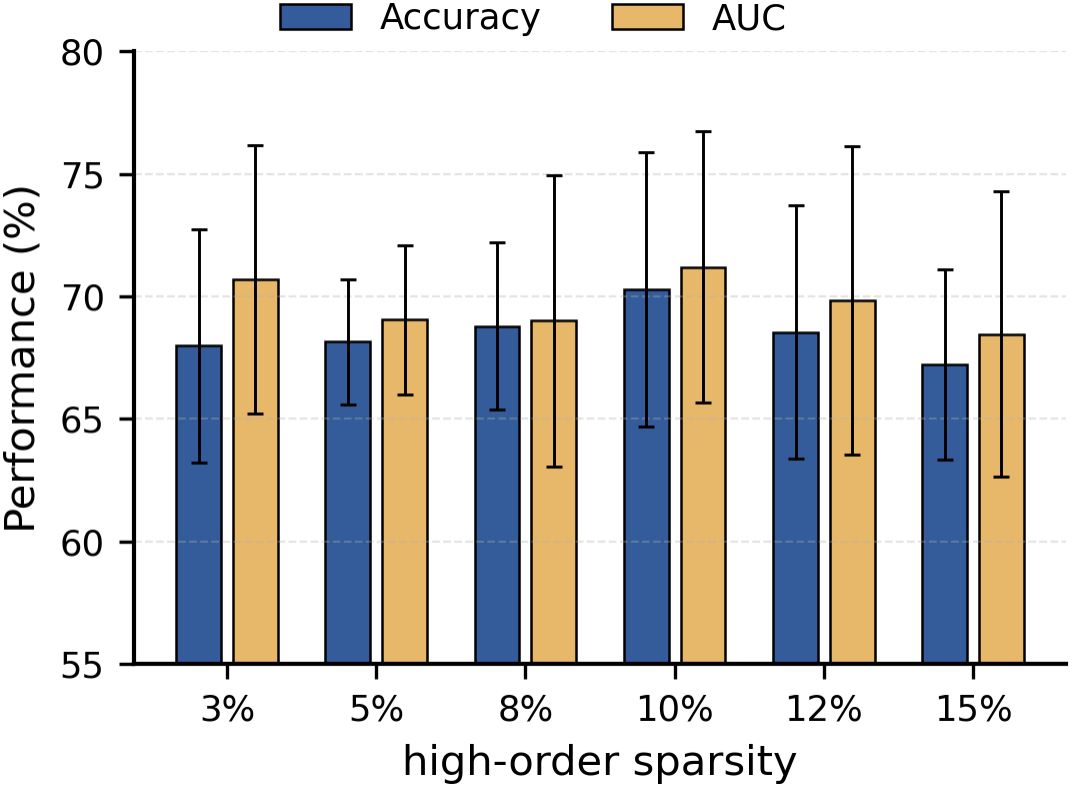
Sensitivity analysis of classification performance under different high-order sparsity settings on the ABIDE-I dataset. The horizontal axis denotes the high-order sparsity setting, and the vertical axis denotes classification performance. Blue and yellow bars denote Accuracy and AUC, respectively, with error bars showing standard deviations.

**Figure 2** shows the sensitivity of classification performance under different high-order sparsity constraints on the ABIDE-I dataset. The results show that performance remains relatively stable mainly within a moderate high-order sparsity range, approximately 8%–12%, rather than across the entire tested interval. When the constraint is overly restrictive, performance exhibits a mild decline due to insufficient incorporation of high-order relational information beyond the low-order consensus. Conversely, excessively permissive constraints tend to introduce weak or noisy long-range connections, which may obscure the discriminative structures learned from lower-order interactions. These results suggest that moderate high-order sparsity achieves a favorable balance between contextual enrichment and noise suppression. Meanwhile, the relatively stable performance within the 8%–12% range indicates that HiNIGAT does not rely on a single finely tuned sparsity value, although appropriate sparsity control is still needed for stable neighborhood construction.

**Figure 3:**
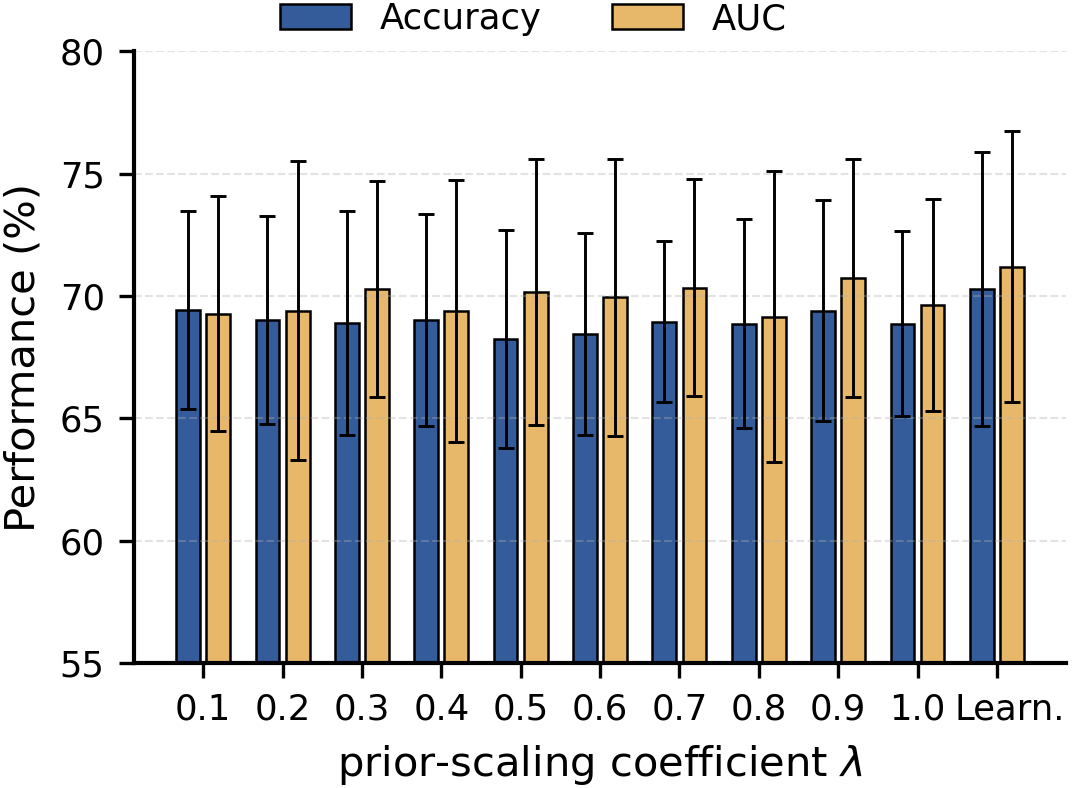
Sensitivity analysis of classification performance under different prior-scaling coefficient settings on the ABIDE-I dataset.

In addition to the sparsity-related parameters in HNC, we further examined the influence of the connectivity-prior scaling coefficient *λ* in MOA. Fixed values *λ* ∈ {0.1,0.2,0.3,0.4,0.5,0.6,0.7,0.8,0.9,1.0} were compared with the learnable *λ* used in the final model. As shown in **Figure 3**, different fixed *λ* values lead to observable performance variations, indicating that the contribution of the connectivity prior should be properly adjusted rather than fixed arbitrarily. The learnable *λ* achieved a more favorable overall balance between accuracy and AUC, especially in terms of AUC, and was therefore adopted in the final model. These results support the effectiveness of using a learnable scaling coefficient to adaptively control the influence of the connectivity prior in attention learning.

### 3.5 Evaluation on the ADHD-200 dataset

To further evaluate HiNIGAT beyond ABIDE-I, we additionally evaluated HiNIGAT on the ADHD-200 dataset. ADHD-200 targets ADHD classification rather than ASD diagnosis, and is therefore used as an additional rs-fMRI-based graph classification task for supplementary evaluation.

Following the same functional brain network construction and model configuration as the ABIDE-I experiments, we evaluated HiNIGAT on ADHD-200 and compared it with representative baseline methods. The corresponding comparison and ablation results are provided in **Supplementary Tables 4**, **5**, and the parameter sensitivity analyses are shown in **Supplementary Figures 3**, **4**. Overall, the results show that HiNIGAT remains competitive on this additional rs-fMRI-based graph classification dataset.

## 4 Discussion

### 4.1 Population-level representation analysis

**Figure 4:**
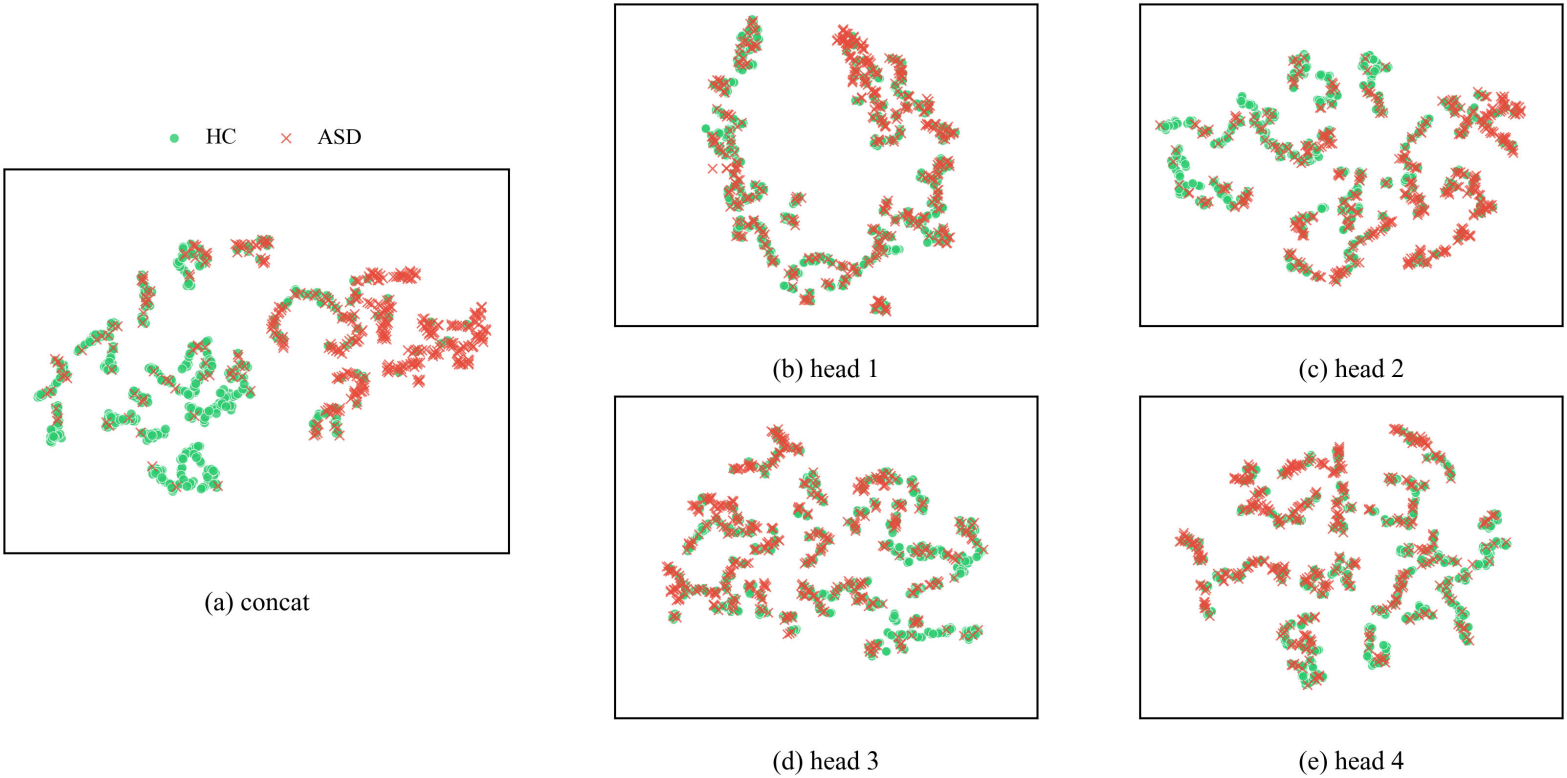
t-SNE visualization of the concatenated multi-head and individual head representations. **(a)** shows the concatenated multi-head representation, and **(b–e)** show the representations from head 1 to head 4, respectively. Green circles denote HC subjects, and red crosses denote ASD subjects.

To evaluate the representation capability of HiNIGAT, we first examine the discriminative properties of the learned embeddings using t-SNE in two-dimensional space, as illustrated in **Figure 4**. Heads 1–4 correspond to representations aggregated from the 1st- to 4th-order neighborhood structures, respectively. As shown in **Figure 4**, the concatenated multi-head feature exhibits more apparent class separation between ASD and HC subjects, forming compact intra-class clusters and distinct inter-class boundaries. In contrast, the single-head embeddings display weaker and more scattered distributions, suggesting that each head captures only partial relational patterns. These results indicate that MOA and BiGF modules effectively integrate complementary information across heads, yielding more discriminative and consistent representations of brain connectivity.

**Figure 5:**
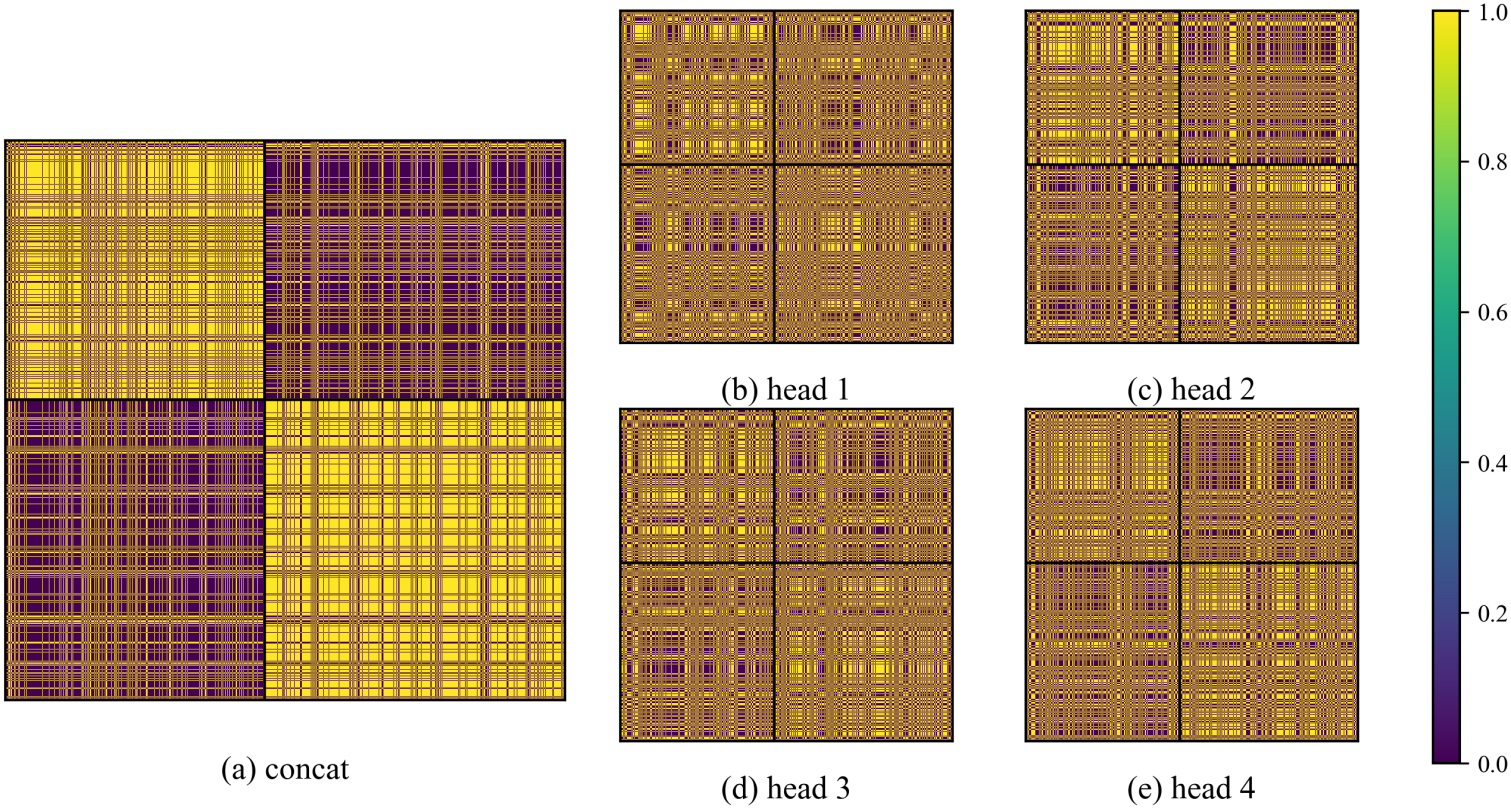
Visualization of the concatenated multi-head and individual head similarity matrices. **(a)** shows the similarity matrix of the concatenated multi-head representation, and **(b–e)** show the similarity matrices from head 1 to head 4, respectively. Brighter colors indicate higher subject-level similarity.

While t-SNE provides an intuitive visualization of embedding separability, it does not explicitly characterize the population-level organization of learned representations. **Figure 5** therefore examines this aspect by visualizing similarity matrices learned across subjects. As shown in **Figure 5**, clear class-specific block-diagonal patterns emerge, indicating improved intra-class coherence and inter-class separation. Notably, the concatenated multi-head representation forms more compact intra-class clusters and clearer separation between ASD and HC subjects than individual heads, suggesting that multi-head fusion effectively enhances global discriminability and within-class consistency. Under supervised learning, the induced population graph progressively enlarges the separation between samples from different classes while reducing distances among samples within the same class. These results highlight the importance of graph structure learning in organizing complex brain network representations and improving classification performance.

**Figure 6:**
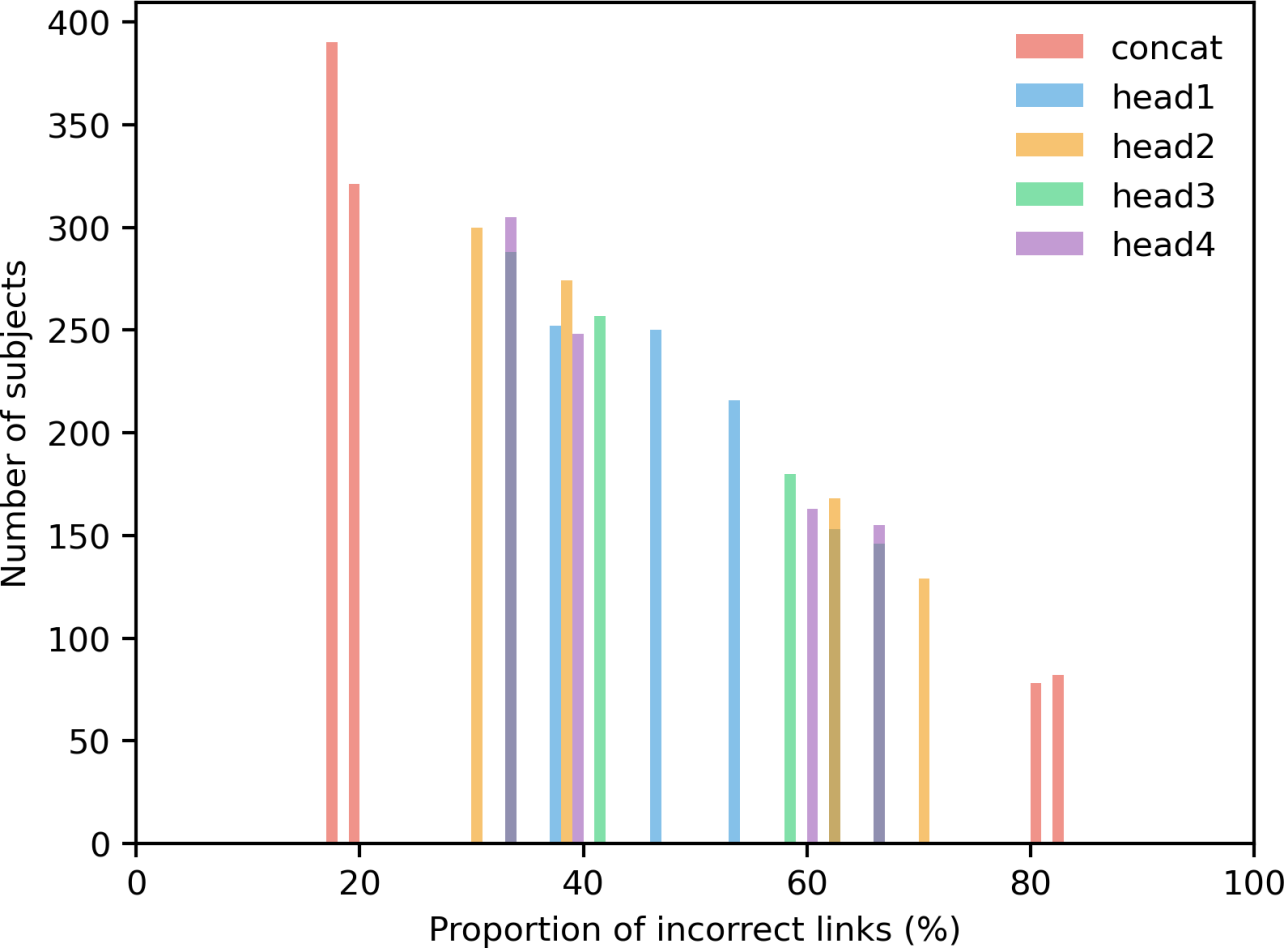
Distribution of incorrect-link proportions across subjects for the concatenated and head-specific representations. The horizontal axis denotes the proportion of incorrect links, and the vertical axis denotes the number of subjects. Different colors correspond to the concatenated multi-head representation and the representations from head 1 to head 4.

While the similarity matrices reflect how subjects cluster in the learned representation space, they do not directly quantify the reliability of the induced population graph. To further examine the quality of the learned graph structure, we measure the proportion of incorrectly predicted links per subject, as shown in **Figure 6**. The concatenated multi-head representation achieves the lowest proportion of incorrect links, whereas individual heads produce higher and more dispersed values. These findings demonstrate that the multi-head fusion mechanism effectively suppresses noise and enhances the semantic consistency of the population graph.

Taken together, these population-level analyses suggest that the hierarchical multi-order design of HiNIGAT is beneficial not only for improving classification performance, but also for organizing subject representations into a more coherent and discriminative population structure. This observation is consistent with the quantitative improvements reported in the Results section and further supports the effectiveness of explicitly modeling and integrating order-specific neighborhood information in FBNs.

### 4.2 Neurobiological interpretability

Beyond predictive performance, it is also important to examine whether the learned model behavior is neurobiologically meaningful. To this end, we further investigate the regional contributions underlying the model’s predictions through ROI-level sensitivity analysis.

Specifically, a gradient-based method was employed to quantify the sensitivity of each ROI node with respect to the model output. Gradients of the loss function with respect to the input features of each node were first computed, then averaged across all samples in the test set. The L2 norm of the resulting gradient vector was taken as the node sensitivity score, with higher values indicating a greater influence on the model’s predictions.

**Figure 7:**
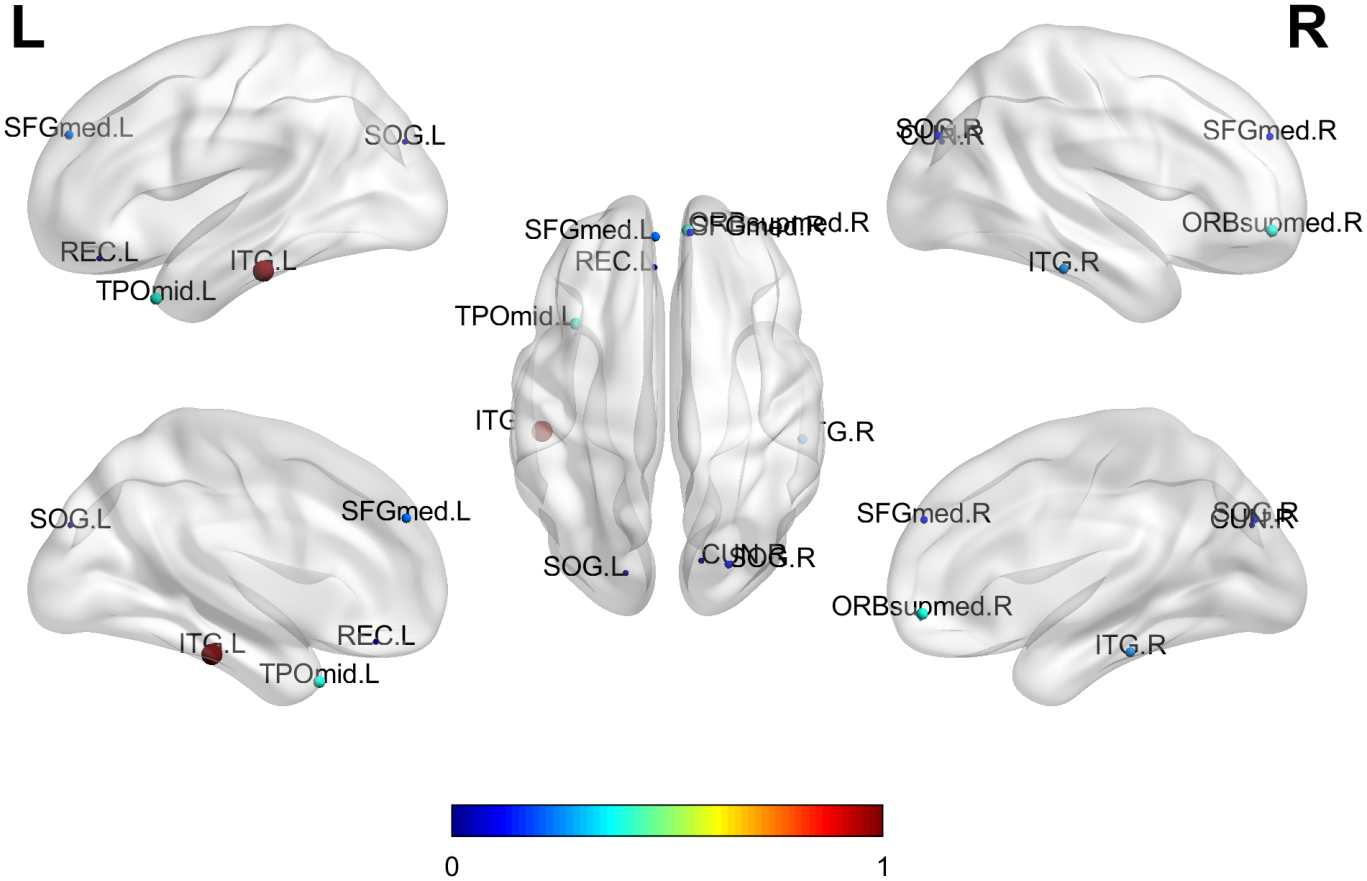
Visualization of the ROI sensitivity analysis. The highlighted brain regions indicate ROIs with high sensitivity scores estimated from the trained HiNIGAT model. Node color and size represent the normalized sensitivity score of each ROI.

The top-ranked sensitive ROIs were visualized on a standard brain surface using the AAL atlas and BrainNet Viewer (33), with node size and color reflecting normalized sensitivity scores (**Figure 7**). The resulting spatial distribution reveals a coherent pattern of discriminative regions. Prominent sensitivity was observed in medial prefrontal and orbitofrontal regions, as well as in several posterior cortical areas. In addition, several temporal and temporo-parietal regions, including the inferior temporal gyrus, middle temporal pole, angular gyrus, and inferior parietal lobule, also exhibited elevated sensitivity scores. Some of these regions overlap with core nodes of the default mode network, particularly posterior cingulate and medial frontal regions, which are commonly associated with self-referential and social cognitive functions. Beyond these core regions, the involvement of orbitofrontal and temporo-parietal association cortices is also compatible with higher-order integrative and affective processing that has been repeatedly implicated in ASD-related brain dysfunction. Notably, orbitofrontal and inferior temporal regions appear among the most sensitive ROIs, highlighting their prominent contribution to the model’s decision-making process.

Overall, the observed region-level sensitivity pattern is broadly consistent with previous neuroimaging findings reporting atypical organization of large-scale functional systems in ASD. From an interpretability perspective, these findings suggest that HiNIGAT does not merely improve classification through opaque feature fitting, but instead captures biologically plausible patterns distributed across multiple functionally relevant brain regions. Although such sensitivity analysis does not establish causal mechanisms, it provides an intuitive bridge between data-driven prediction and existing neuroscientific knowledge, thereby improving the interpretability of the proposed framework.

### 4.3 Limitations and conclusions

Despite the encouraging results, several limitations should be acknowledged. First, the current evaluation is still mainly based on publicly available rs-fMRI datasets with limited sample size and substantial inter-site heterogeneity. Although repeated site-grouped cross-validation was adopted, differences in data acquisition, subject composition, and preprocessing pipelines may still influence the learned graph structures and classification performance. Further validation on independent external datasets is therefore needed to assess the robustness and generalizability of HiNIGAT.

Second, although the proposed framework improves interpretability through order-specific attention and ROI-level sensitivity analysis, the resulting importance patterns should still be interpreted cautiously. The learned attention coefficients and sensitivity scores primarily reflect statistical relevance within the model rather than direct neurobiological causality. Therefore, the current interpretability analysis is more suitable for supporting population-level investigation than for individual-level clinical decision-making.

Third, HiNIGAT is currently developed under a fixed brain parcellation scheme and subject-specific FBN construction pipeline. Its robustness under different atlases, functional connectivity estimation methods, graph thresholding strategies, and sparsity configurations remains to be further examined. In addition, the strict order-specific construction in HNC may still simplify the overlapping roles of functional connections across different topological orders. More flexible order modeling strategies, such as continuous order-aware weighting or learnable order-aware fusion, therefore remain worth further investigation.

Finally, HiNIGAT models rs-fMRI mainly through static ROI-level functional connectivity and therefore does not explicitly capture temporal-frequency dynamics in the original BOLD signals. ASD-related abnormalities may also involve time-varying connectivity patterns and nonlinear oscillatory features. Recent studies on Fourier-attention (34), wavelet-attention (35, 36), and stability-theoretic modelling (37–39) provide useful directions for incorporating temporal-frequency features as node attributes or as an additional branch in future multimodal fusion frameworks.

In summary, the proposed HiNIGAT framework offers an effective and interpretable approach for functional brain network analysis in ASD. Future work will further evaluate the proposed framework on additional independent datasets, extend it to more heterogeneous and multimodal settings, and investigate its applicability to broader brain network classification problems beyond ASD.

## Data Availability

The original contributions presented in the study are included in the article/**Supplementary Material**. Further inquiries can be directed to the corresponding authors.
