# Supplementary material for: Hierarchical neighbor integration graph attention network for autism spectrum disorder diagnosis
Source: Front Psychiatry. 2026 Jul 20;17:1846996. doi: 10.3389/fpsyt.2026.1846996 (PMC13430121; doi:10.3389/fpsyt.2026.1846996)
Supplement: Supplementary file 1 [file DataSheet1.pdf]

Supplementary Material

1 ABIDE-I LOSO RESULTS

In addition to the repeated site-grouped 10-fold cross-validation experiments reported in the main text, we conducted a leave-one-site-out (LOSO) evaluation on ABIDE-I as a supplementary site-wise generalization analysis. In this setting, each acquisition site was held out in turn as the test set, and all remaining sites were used for training. The comparison and ablation results are reported as mean ± standard deviation across held-out acquisition sites in Tables S1 and S2, respectively. The corresponding sensitivity analyses for the high-order sparsity constraint and the prior-scaling coefficient are shown in Figures S1 and S2. Overall, these supplementary LOSO results show that HiNIGAT maintains competitive site-wise performance on ABIDE-I. The ablation results further show that the complete HiNIGAT model achieved the best accuracy, precision, F1-score, and AUC, although some ablated variants obtained higher recall. This suggests that the complete model provided a more favorable overall balance under the LOSO setting. The sensitivity results further illustrate the performance variations under different sparsity and prior-scaling settings.

Table S1. LOSO performance comparison of various methods on the ABIDE-I dataset.

Method	Accuracy	Precision	Recall	F1-score	AUC
SVM	62.93 ± 7.27	61.29 ± 15.09	60.94 ± 16.04	58.82 ± 10.75	68.69 ± 10.65
MLP	61.38 ± 6.07	57.92 ± 12.08	58.97 ± 15.34	57.12 ± 10.02	64.21 ± 7.82
BrainGNN	67.57 ± 5.52	67.28 ± 21.33	58.03 ± 25.64	58.20 ± 17.99	65.41 ± 9.00
GCN	66.73 ± 6.15	67.04 ± 14.34	60.60 ± 17.63	60.95 ± 10.51	63.58 ± 8.54
GraphSAGE	69.37 ± 5.64	69.57 ± 14.31	$\mathbf{65.21 \pm 23.24}$	63.16 ± 14.70	66.93 ± 9.47
GAT-2L	68.48 ± 6.48	68.47 ± 15.10	58.98 ± 22.73	60.58 ± 14.62	64.96 ± 12.04
GAT-4L	68.47 ± 5.94	69.80 ± 14.54	60.72 ± 23.05	60.23 ± 17.59	65.97 ± 10.20
GAT-6L	67.66 ± 6.81	72.17 ± 16.22	58.13 ± 21.68	59.37 ± 15.80	66.37 ± 9.66
MixHop	70.99 ± 7.27	71.24 ± 12.60	65.12 ± 19.56	65.26 ± 13.44	68.20 ± 10.15
SIGN	67.05 ± 4.00	65.22 ± 9.95	60.38 ± 18.79	60.61 ± 11.94	65.55 ± 7.14
HiNIGAT	$\mathbf{71.91 \pm 6.85}$	$\mathbf{72.69 \pm 12.44}$	65.19 ± 12.45	$\mathbf{67.28 \pm 8.52}$	$\mathbf{71.15 \pm 9.21}$

Table S2. LOSO ablation study of HiNIGAT on the ABIDE-I dataset.

Method	Accuracy	Precision	Recall	F1-score	AUC
w/o MOA	67.89 ± 5.97	65.40 ± 14.48	71.72 ± 18.09	65.84 ± 8.93	67.58 ± 9.26
w/o HO	67.81 ± 6.85	63.41 ± 12.03	71.82 ± 23.19	64.83 ± 14.03	68.47 ± 10.53
w/o OS	65.93 ± 7.95	61.64 ± 20.59	61.70 ± 27.63	58.40 ± 19.51	63.81 ± 13.14
w/o SOS	67.13 ± 7.04	59.37 ± 18.74	70.91 ± 23.44	63.35 ± 18.39	63.99 ± 12.10
w/o TW	66.47 ± 6.96	61.45 ± 10.47	$\mathbf{72.15 \pm 14.28}$	65.51 ± 9.39	65.81 ± 8.46
w/o BiGF	65.20 ± 8.67	59.45 ± 10.59	71.29 ± 19.97	63.65 ± 12.74	65.45 ± 9.75
w/o TD	66.65 ± 5.41	59.48 ± 18.28	65.91 ± 24.09	61.00 ± 18.63	64.72 ± 11.73
w/o BU	68.20 ± 6.51	60.97 ± 18.17	69.78 ± 26.18	63.18 ± 19.08	65.66 ± 13.45
HiNIGAT	$\mathbf{71.91 \pm 6.85}$	$\mathbf{72.69 \pm 12.44}$	65.19 ± 12.45	$\mathbf{67.28 \pm 8.52}$	$\mathbf{71.15 \pm 9.21}$

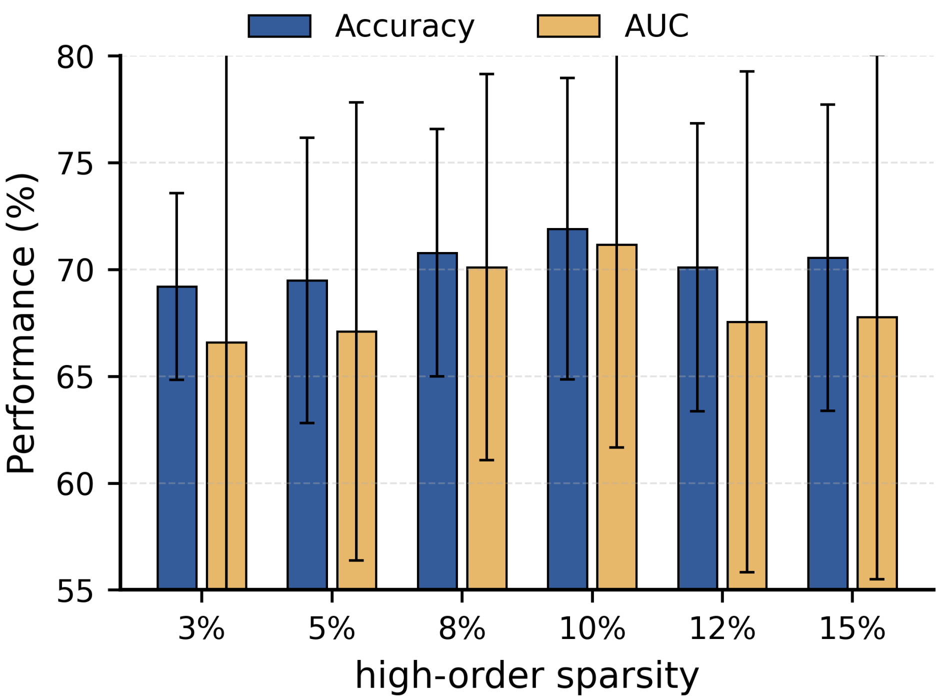

Figure S1. Sensitivity analysis of classification performance for different high-order sparsity constraints under the ABIDE-I LOSO setting.

2 ADHD-200 DATASET AND EVALUATION PROTOCOL

To further evaluate HiNIGAT beyond the ABIDE-I dataset, we additionally conducted experiments on the ADHD-200 dataset. ADHD-200 targets ADHD classification rather than ASD diagnosis, and is therefore used as an additional rs-fMRI-based graph classification task for supplementary evaluation. Following prior studies, we used the preprocessed data released by the Neuro Bureau ADHD-200 Global Competition. All ADHD-200 data were preprocessed using the Athena pipeline, which applies standard preprocessing steps including slice timing correction, head motion correction, spatial normalization, temporal band-pass filtering, and spatial smoothing. Consistent with the quality-control-based subject-screening procedure reported by Dai et al. (2012), the screening was performed at the subject level based on diagnostic-label availability and available resting-state fMRI quality-control information. Subjects without available diagnostic labels or usable rs-fMRI quality-control records were excluded. Subjects belonging to the ADHD-Hyperactive type were also excluded because of the limited sample size. In addition, Pittsburgh and WashU were excluded from the ADHD/HC classification experiments because these two sites were severely imbalanced toward healthy controls after screening. After this screening, five sites were retained for evaluation, including NYU, Peking, OHSU, KKI, and NeuroIMAGE (NI). The final ADHD-200 subset contained 521 subjects, including 154 ADHD patients and 367 healthy controls (HCs). The demographic information is summarized in Table S3.

The functional brain network construction and model configuration were kept consistent with those used in the ABIDE-I experiments described in the main text. Since only five ADHD/HC sites were retained after screening, we adopted a leave-one-site-out evaluation for ADHD-200. Class-weighted cross-entropy was used consistently in the ADHD-200 experiments to account for the imbalanced ADHD/HC distribution in the retained subset.

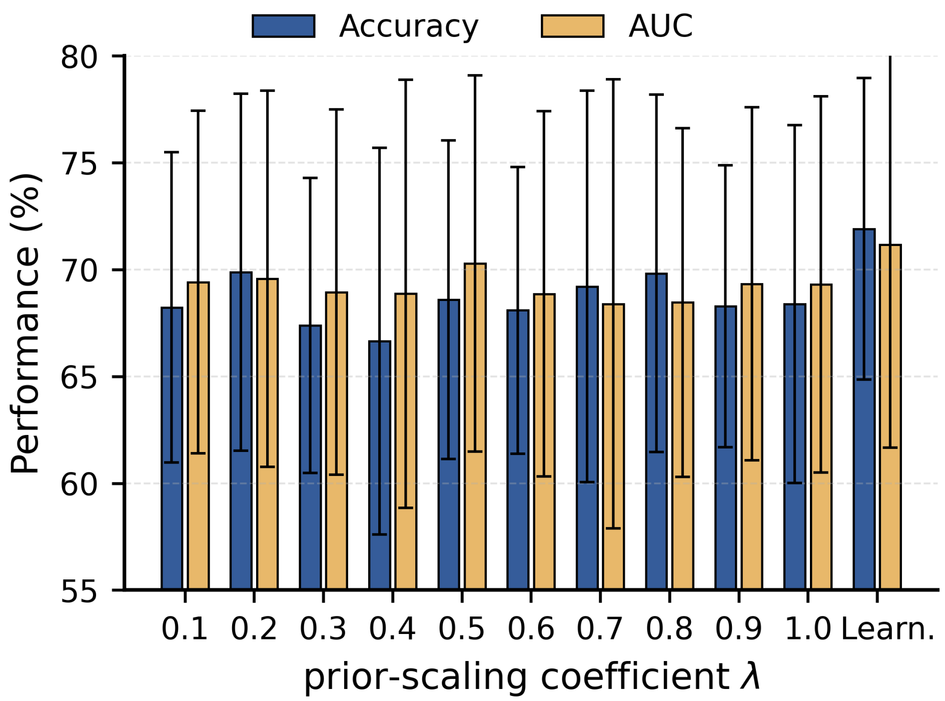

Figure S2. Sensitivity analysis of classification performance for different prior-scaling coefficient settings under the ABIDE-I LOSO setting.

3 ADHD-200 LOSO RESULTS

The results on the ADHD-200 dataset are shown in Tables S4-S5, and Figures S3-S4. As shown in Table S4, HiNIGAT achieved the best accuracy, F1-score, and AUC among the compared methods on this independent dataset. Although some baseline methods obtained higher precision or recall, their performance was less balanced across the remaining metrics. Table S5 further reports the ablation results. The complete HiNIGAT model achieved the best accuracy, F1-score, and AUC, whereas some ablated variants showed higher single-metric precision or recall but weaker overall balance. In addition, Figure S3 shows the performance variation under different high-order sparsity constraints, and Figure S4 shows the results under different prior-scaling coefficient settings. Overall, these results provide supplementary descriptive evidence for the applicability of HiNIGAT under the ADHD-200 LOSO setting.

Table S3. Demographic information of the ADHD-200 dataset.

Site	ADHD		HC	
	Age	Gender (M/F)	Age	Gender (M/F)
NYU	10.8 ± 2.6	44/11	12.8 ± 3.0	38/41
Peking	11.6 ± 1.8	37/0	11.4 ± 1.9	78/58
OHSU	8.9 ± 1.3	12/4	9.3 ± 1.4	24/26
NI	17.0 ± 2.6	26/2	18.6 ± 3.1	11/25
KKI	9.8 ± 1.6	10/8	10.3 ± 1.3	38/28
All sites	11.8 ± 3.4	129/25	11.9 ± 3.3	189/178

Table S4. LOSO performance comparison of various methods on the ADHD-200 dataset.

Method	Accuracy	Precision	Recall	F1-score	AUC
SVM	66.49 ± 4.68	45.59 ± 15.25	35.41 ± 7.48	37.73 ± 4.03	60.45 ± 1.67
MLP	63.39 ± 7.70	43.22 ± 18.32	35.63 ± 13.67	35.06 ± 7.67	60.39 ± 4.54
BrainGNN	66.50 ± 8.45	50.57 ± 17.76	57.19 ± 17.67	49.25 ± 11.25	66.80 ± 7.77
GCN	63.94 ± 5.11	44.75 ± 12.95	64.61 ± 14.89	50.44 ± 7.84	67.53 ± 6.96
GraphSAGE	58.82 ± 11.20	43.25 ± 18.56	72.10 ± 23.14	49.98 ± 10.64	59.46 ± 18.12
GAT-2L	54.48 ± 14.15	40.81 ± 15.44	$\mathbf{74.97 \pm 14.32}$	49.36 ± 9.17	63.96 ± 10.66
GAT-4L	60.78 ± 7.28	42.20 ± 13.93	68.75 ± 11.38	50.34 ± 10.32	64.80 ± 5.21
GAT-6L	62.08 ± 11.07	43.84 ± 14.93	72.75 ± 17.34	52.44 ± 12.10	66.63 ± 6.48
MixHop	62.12 ± 1.80	44.66 ± 19.77	58.01 ± 20.47	45.23 ± 10.52	64.71 ± 9.48
SIGN	63.24 ± 6.57	$\mathbf{50.96 \pm 26.79}$	47.63 ± 20.38	40.58 ± 12.08	64.66 ± 9.00
HiNIGAT	$\mathbf{68.68 \pm 3.65}$	46.82 ± 14.08	65.47 ± 17.96	$\mathbf{53.63 \pm 13.26}$	$\mathbf{68.39 \pm 11.32}$

Table S5. LOSO ablation study of HiNIGAT on the ADHD-200 dataset.

Method	Accuracy	Precision	Recall	F1-score	AUC
w/o MOA	62.15 ± 4.52	44.33 ± 16.94	$\mathbf{70.47 \pm 19.55}$	50.89 ± 10.54	65.32 ± 9.56
w/o HO	64.85 ± 4.48	43.97 ± 12.97	66.00 ± 22.09	50.44 ± 12.12	63.50 ± 8.50
w/o OS	64.79 ± 7.08	43.87 ± 15.13	65.28 ± 17.82	50.81 ± 13.66	64.16 ± 9.75
w/o SOS	60.43 ± 7.31	41.14 ± 16.51	68.62 ± 12.20	50.00 ± 14.04	63.82 ± 10.15
w/o TW	67.82 ± 6.15	45.83 ± 13.78	64.37 ± 16.36	52.72 ± 13.46	65.29 ± 8.16
w/o BiGF	65.77 ± 7.13	44.69 ± 12.48	62.57 ± 16.49	50.77 ± 11.07	65.14 ± 9.44
w/o TD	66.34 ± 8.16	46.20 ± 12.33	64.44 ± 13.99	52.43 ± 9.82	65.51 ± 8.42
w/o BU	68.17 ± 8.31	$\mathbf{47.79 \pm 14.49}$	64.40 ± 15.71	53.47 ± 12.19	66.06 ± 9.47
HiNIGAT	$\mathbf{68.68 \pm 3.65}$	46.82 ± 14.08	65.47 ± 17.96	$\mathbf{53.63 \pm 13.26}$	$\mathbf{68.39 \pm 11.32}$

REFERENCES

Dai D, Wang J, Hua J, He H. Classification of ADHD children through multimodal magnetic resonance imaging. *Frontiers in Systems Neuroscience* **6** (2012) 63.

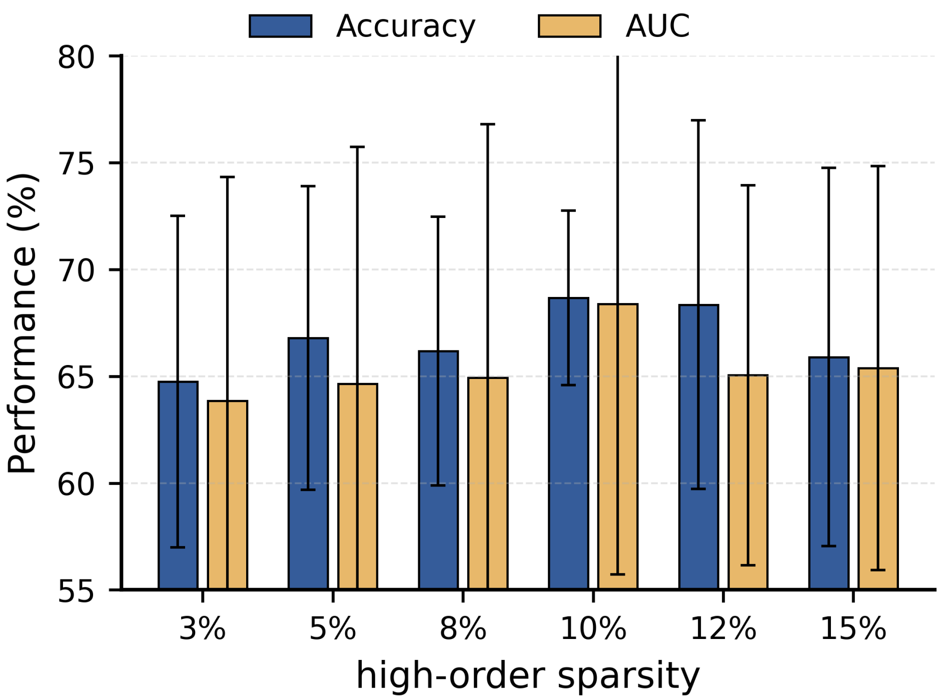

Figure S3. Sensitivity analysis of classification performance for different high-order sparsity constraints under the ADHD-200 LOSO setting.

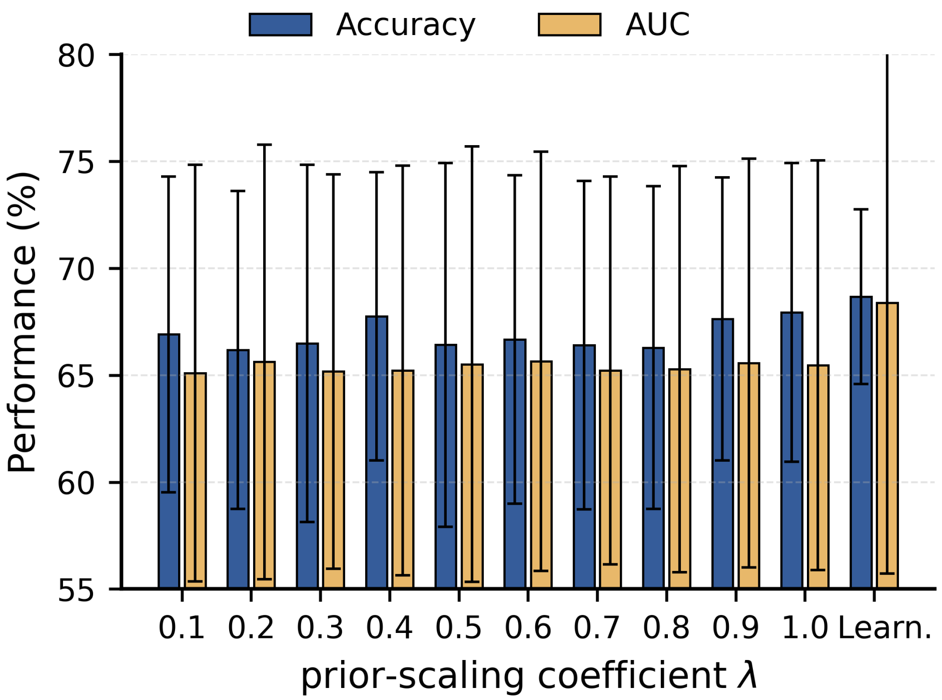

Figure S4. Sensitivity analysis of classification performance for different prior-scaling coefficient settings under the ADHD-200 LOSO setting.
